# Dynamically Cross-Linked Tannin as a Reinforcement of Polypropylene and UV Protection Properties

**DOI:** 10.3390/polym11010102

**Published:** 2019-01-09

**Authors:** Jingjing Liao, Nicolas Brosse, Antonio Pizzi, Sandrine Hoppe

**Affiliations:** 1 LERMAB, University of Lorraine, Boulevard des Aiguillettes BP 70239, 54506 Vandœuvre-lès-Nancy, France; jingjing.liao@univ-lorraine.fr (J.L.); antonio.pizzi@uinv-lorraine.fr (A.P.); 2 LRGP, University of Lorraine, 1, Rue Grandville, BP 451, 54001 Nancy CEDEX, France; sandrine.hoppe@univ-lorraine.fr

**Keywords:** tannin, dynamic cross-linked, polypropylene, UV weathering

## Abstract

Tannins were used as reinforcing components for polypropylene with anti-UV properties via dynamic curing extrusion. The influence of cross-linked tannins in different weight fraction and their anti-UV capacity on morphological, mechanical, rheological, crystallize and thermal properties were studied. The experimental results indicated that the cross-linked tannins improve Young’s modulus, crystallinity, and thermal stability and reinforce the internal network of polypropylene. After UV accelerated weathering, polypropylene had fewer surface cracks, lower carbonyl index, fewer crystallinity decreases and less mechanical properties loss with increasing tannin content.

## 1. Introduction

Over the past few decades, polymer composites have been widely studied due to their advantageous combined properties over single-component polymers. With the increasing focus on environmental awareness, considerable efforts have been made to develop composites that contain some fraction of sustainable biopolymers due to their renewable, biodegradable, chemical reactivity and physicochemical properties [1]. Among those composites, polypropylene (PP) is one of the most important and widely used composite matrix because of its low production cost, design flexibility, low density, and recyclability [2]. The combination of PP and biopolymers show many advantages, including low cost, availability of renewable natural resources, biodegradability and good mechanical properties [3,4,5,6]. For example, cellulose and cellulosic materials are widely used as a reinforcement in PP-based composites. However, PP is well known to be highly sensitive to UV light and oxygen compared with other polyolefins. The UV exposure results in photodegradation, causing color changes, surface cracking and ultimately lower mechanical properties [7].

The photodegradation mechanisms of PP is mainly initiated by UV irradiation, creating broken bonds and free radicals in the polymer (Equation (1)). Oxygen combines with free-radical species to create peroxy radicals which are reactive with polymer chain and hydroperoxides which are reactive to create hydroxyl and alkoxy radicals (Equations (2) and (3)) [8,9]. Additives with the capacity to absorb the UV energy or scavenge free-radical species or peroxides can physically or chemically prevent or interrupt the degradation processes [7]. For this reason, polymers like phenolics antioxidant are considered as effective additives of PP due to their free radical scavenging capacity.

(1) $$RH\overset{hv}{\to }R$$

(2) $$R\;\cdot +O_{2}\to R\;R\;\cdot +\mathrm{RH}\to ROOH+R$$

(3) ROOH→· OH+RO

**Figure 1:**
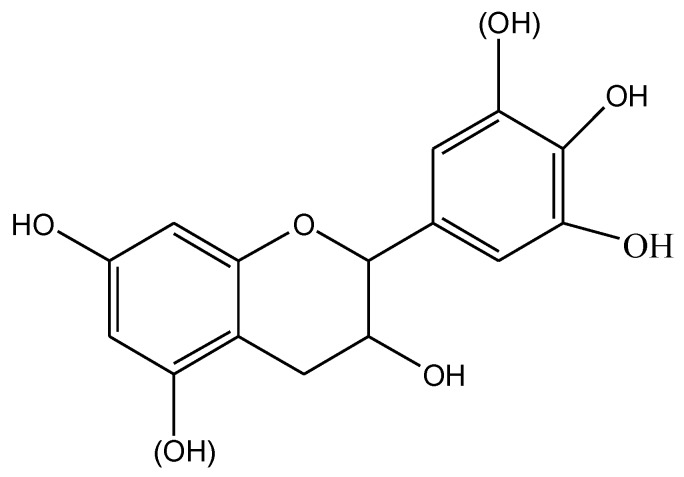
Structure of flavonoid unit of condensed tannins.

Recently, natural phenolic compounds are gaining attention driven by environmental concern. Lignin is the most widely studied one for PP based composites [10]. Its reinforcing [11], antioxidant [12,13], or stable function [14] have been extensively reported. However, reports concerning the utilization of tannins in mixture with thermoplastic resins are scarce. Tannins are the fourth most abundant sustainable biopolymers from plants (after cellulose, hemicellulose, and lignin), present in soft tissues of woody plants like leaves, needles, and bark [15]. Tannins are commonly divided into hydrolyzable tannins and condensed tannins. Condensed tannins which are mainly composed of flavonoid units (Figure 1) are widely distributed in nature and constitute more than 90% of the total world production of commercial tannins [16]. Because of their high chemical reactivity, tannins have played an important role in thermosetting systems for several decades, such as tannin-based adhesives for wood bonding [17,18,19], tannin-based foam material [17]. Moreover, the structure of flavonoids presents suitability for UV protection and antioxidation in plants and this has led to the application to prevent human aging [18]. 

According to some recent works, the addition of flavonoid structure (chrysin, quercetin, and silibinin) on PP, yielded a good stabilization against UV radiation [19]. Ambrogi and co-workers also verified the stabilization property of tannins rich product and other phenol by-products on PP according to the experimental results of thermogravimetric analysis and oxidative induction time measurement, which is similar as a commercial phenolic antioxidant [20]. In addition, tannins used as co-stabilizer for poly (vinyl chloride) were investigated and have shown an improvement in thermal stabilization and thermal stability and did not cause negative impacts during production process [21]. Some researchers demonstrated that tannins are effective short-term stabilizers for polyethylene films, reducing the impact of thermo-oxidative and UV degradation [22]. Furthermore, tannin also is a functional component of the polyvinyl alcohol (PVA) composite membrane with antioxidant and UV-protective properties [23]. However, tannins like other biopolymers containing hydrophilic hydroxyl groups result in incompatibility with a hydrophobic polymer and therefore deteriorate the mechanical properties of the final product when increasing their proportion. As a result, the chemical modification of biopolymers is generally considered as the most effective technique to enhance the biopolymer-matrix adhesion [24,25]. Moreover, this chemical modified biopolymer reduces not only the hydrophilicity but also develops in some cases functional property. For example, tannin-based epoxy resin (with 5, 10 or 20 phr) can provide enhanced dynamic thermal and process stability and improved the rheological properties on PVC [26]; acetylated tannin or esterified tannin exhibited improved miscibility with hydrophobic plastic matrix (e.g., poly(butylene succinate), PP, aliphatic polyester, poly(lactic acid)), and UV stability [27,28,29]. The addition of compatibilizers is also an effective pathway to improve biopolymers and plastics matrix by the formation of bonds, maleic anhydride grafted polypropylene (MAPP) being one of the most important compatibilizers for a PP matrix due to the high reactivity of maleic anhydride [30,31]. The utilization of maleic anhydride functionalized polymer has shown better compatibility for polyethylene–tannin films which further enhanced their thermal properties [22]. The goal of these interfacial modification methods is to minimize the hydroxyl groups of biopolymers to reduce their polar difference, therefore, to enhance biopolymers-matrix adhesion. On this basis, a dynamic vulcanization process might be a good technique to decrease their hydrophilicity. This process has been successfully applied to dynamically crosslink thermoplastic resin with thermoset resin system. For example, dynamically cross-linked PP/epoxy composite [32] and PP/novolac composite [33] compatibilized with MAPP were reported to exhibit higher modulus, stiffness as well as thermal stability compared to neat PP. 

In the present work, tannin was dynamically cross-linked with hexamethylenetetramine (hexamine) within a twin-screw extruder to reinforce a PP matrix. Hexamine has been described as a cross-linker for tannin resins usable for wood bonding [17]. Meanwhile, 5% MAPP have been used as a compatibilizer. The influence of dynamically cross-linked tannin (TH) in different weight fraction on morphological, mechanical, rheological and thermal properties were evaluated. Moreover, the anti-UV properties has been assessed by the changes of mechanical property, surface morphology, crystallinity, rheological behavior after UV accelerated weathering test.

## 2. Materials and Methods

### 2.1. Materials

The PP was purchased from TOTAL with a melt flow index of 25 g/min according to the standard test method ISO 3146 and a melting temperature of 165 °C. The maleic anhydride grafted polypropylene, Licocene^®^ PP MA 6252 granules wax (grafting rate 1%), were supplied by Clariant (Muttenz, Switzerland) with a viscosity (approx.) 100 mPa·s at 170 °C. Mimosa tannins were purchased from Silva Chimica, in Mondovi, Italy. The hexamethylenetetramine 99% (hexamine) and sodium hydroxide were purchased at Acros Organics (Illkirch-Graffenstaden, France) and VWR Company (Radnor, PA, USA), respectively.

### 2.2. Preparation of PP/TH Composites

Tannin was pre-reacted with hexamine in a stirred reactor and dried by a spray dryer (BUCHI mini spray dryer B-290, Flawil, Suisse) before blended with PP. The 100 parts of mimosa tannin extracts were dissolving in 100 parts of water and 12 parts of NaOH were mixed for adjusting its pH to 10. And tannin solutions were heated to 80 °C and then six parts of hexamine were added to the mixture for 30 min. All the compounds were added based on the weight of the dry tannin. The spray dried pre-reacted tannin-hexamine(TH) was characterized by FTIR (Figure S2 and Table S3).

PP blend with 5%, 10%, 15%, 30% pre-reacted tannin-hexamine (note as 5TH, 10TH, 15TH, and 30TH, respectively) were carried out in a twin-screw extruder (Thermo Scientific^TM^ Process 11, Villebon-sur-Yvette, France) with a screw rotation speed of 200 rpm at 190 °C. The extruder had a screw diameter 11 mm and a length-to-diameter ratio L/D = 40. The screw profile is composed of three mixing zones with different configurations of kneading blocks for promoting dispersive and distributive mixing. The residence time was approximate 2 min 30 s. PP powder, pre-reacted tannin-hexamine and PPMA in different weight proportion were well blended in a baker before injecting into the extruder. Tannin–hexamine were expected to be cured under the pressure and temperature of the the extruder. Unreacted species and reaction by-products were removed by a highly efficient vacuum in zone 6. 

### 2.3. Characterization

UV accelerated weathering test was carried out by a QUV accelerated weathering tester (SOLAR EYE, Q-Panel Lab Products, Saarbrucken, Germany). The specimens were exposed under continuous irradiation with an irradiance intensity of approximately 0.68 W/m^2^/nm at λ = 340 nm for one week (168 h) at 60 °C. For each batch of specimens, four replicates were used to ensure the repeatability. The anti-UV properties of TH were evaluated by the changes in surface morphology, mechanical property, rheological behavior, crystallinity and surface chemical property.

The dispersion of TH was observed by Dino-lite premier digital microscopy (AM-7013MZT, New Taipei City, Taiwan, China) with a software DinoCapture 2.0. The morphology of UV exposed surface was characterized by a scanning electron microscopy (SEM, Hitachi S4800, Los Angeles, CA, USA) with an acceleration voltage of 15 kV. All the samples were sputter coated with a thin layer of gold.

The tensile test was performed at room temperature with standard dog-bone shaped (ISO 527, type 1A) tensile test specimens, which was molded by a micro-injection molder (Xplore, Sittard, the Netherlands), on an Instron tensile testing machine (model 5569) equipped with a 50 kN load cell, operated according to EN ISO 527:1996. The cross-head speed used was of 10 mm/min. All the samples were measured four times and the the average value was calculated.

The rheological property was performed using a rheometer (TA instrument, G2 ARES, New Castle, DE, USA) with parallel-plate geometry (25 mm diameter, 1 mm gap) with the protection of nitrogen under 180 °C. Frequency sweeps between 0.05 to 100 rad/s were carried out at 10% strain in the linear viscoelastic region (LVE).

Thermogravimetric analysis (TGA) was carried out in a METTLER TOLEDO. The samples with a total weight in the 5–10 mg range were scanned in the range of 30–600 °C with a heating rate 10 °C/min in an air atmosphere (50 mL/min). The obtained data were analyzed by STARe evaluation software (version 10.0).

The Differential scanning calorimetry (DSC, METTLER TOLEDO) was employed to measure the melting temperature (*T_m_*) and crystallinity temperature (*T_c_*) of samples in the second heat-cool-heat circle with a scan rate of 10 °C/min within the temperature range of −50 °C to 220 °C. The measurements were using aluminum crucibles with a total sample weight 5.00 ± 1 mg under a nitrogen atmosphere (50 mL/min). Values for melting temperatures (*T_m_*) and enthalpy of melting (*H_m_*) were analyzed by STARe evaluation software. The percentage of crystallinity *X_c_* was estimated by the following equation:

(4) $$X_{c}\left(\%\right)=\frac{100\times H_{m}}{\Delta H_{m}^{0}\times w}$$

where *X_c_* is the crystallinity (%), $\Delta H_{m}^{0}$ is the enthalpy of melting 100% crystallized PP, which is equal to 207 J/g [34], *H_m_* is the enthalpy required for melting each sample, and w is the weight fraction of PP in blends.

The Fourier Transform Infrared (FT-IR) spectra of the exposed surface of aged PP and PP/TH specimens measured by a NICOLET 6700 FT-IR spectrometer attenuated total reflection (ATR) mode for 16 scans in the range 4000–400 cm^−1^ with a resolution of 4 cm^−1^. The curves were normalized, and peaks were analyzed without smoothing the data. The degradation of composites was characterized by carbonyl index (*CI*) [34]. The calculation equation as follows:

(5) $$CI=\frac{A_{c}}{A_{r}}$$

where *A_c_* is the area of the carbonyl absorption band (1670–1820 cm^−1^, C=O). *A_r_* is the area of C-H stretch from CH_3_ (2760–3020 cm^−1^). The latter one was used as a reference band because it was minimally affected by UV irradiation. In addition, for a more precise evaluation, the calculated carbonyl area *A_c_* of each sample has been minus the area before aging, considering that tannin has carbonyl groups in their structure. 

## 3. Results and Discussion

### 3.1. The Effects of Dynamically Cross-Linked Tannin and Its Reinforcing Function on PP

#### 3.1.1. Morphology

**Figure 2:**
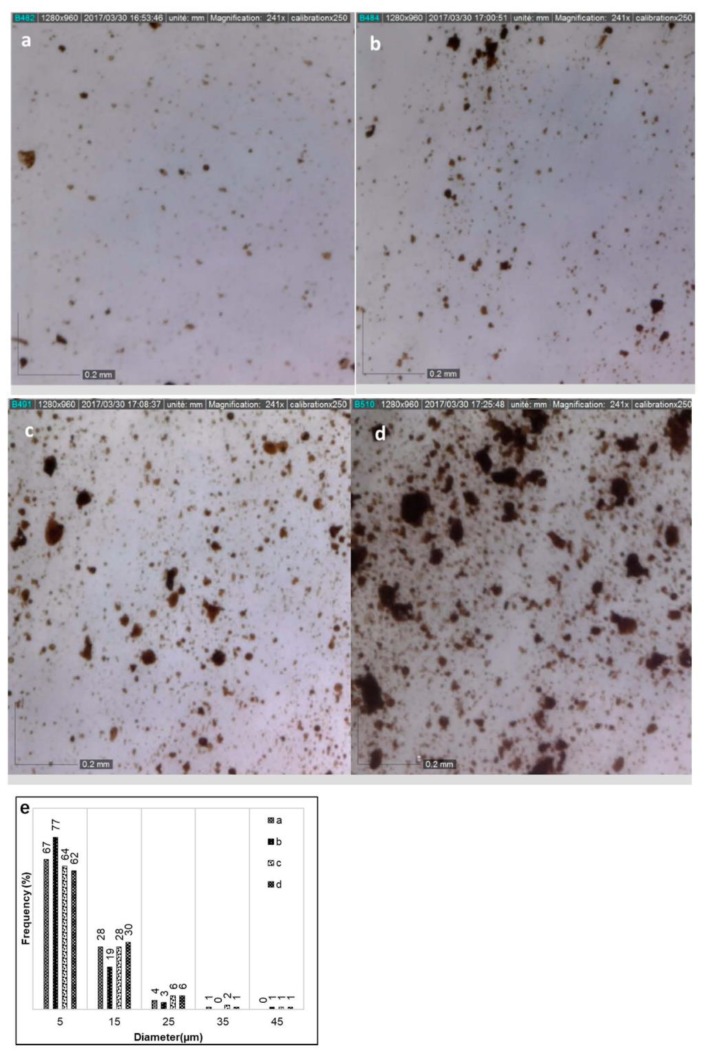
Micrographs of PP/TH composites: (**a**) 5TH, (**b**) 10TH, (**c**)15TH, (**d**) 30TH, (**e**) particles size distributions.

The composites have been prepared by twin extrusion of PP blend with 5% to 30% of a pre-reacted tannin-hexamine mix (TH). The morphology of PP/TH composites was observed by optical microscopy and SEM. SEM micrographs (Supplementary Materials, Figure S1) of their fracture surface are smooth and the cross-linked tannins are difficult to observe, indicating fine cross-linked tannin particles well dispersed in the PP matrix. Similar results can be found in dynamic cured a PP/phenolic resin and PP/epoxy blends [32,35]. At the higher TH content (30%) one can observe a rough region marked with a white circle, which may be due to the agglomeration of cross-linked tannin. To further identify the morphology of PP/TH composites, film shape materials (0.6 mm) were prepared by micro-compounder and observed by optical microscopy. The particles size distributions of PP/TH composites can be observed. Figure 2a–d shows the micrographs of PP films with different cross-linked tannin content. Figure 2e gives the particle size distribution analyzed by software Image J. From these micrographs, the cross-linked tannin appears to be homogeneously dispersed as particles within the PP matrix. These particles are generally fine in the composites with 5% cross-linked tannin (Figure 2a), while TH agglomeration starts to occur with increasing tannin content (Figure 2b–d). However, the bar chart (Figure 2e) confirms the good dispersion of cross-linked tannin and 90% of particles are in the average diameter range of 5–15 μm.

#### 3.1.2. Tensile Properties

**Figure 3:**
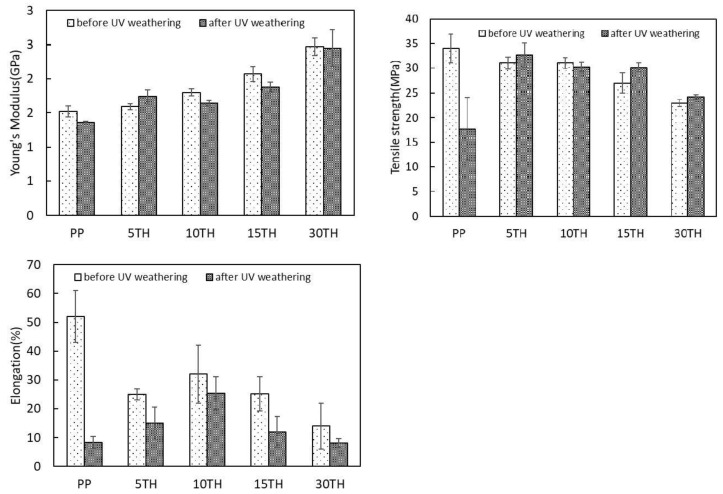
The tensile property of PP and PP/TH composite before and after UV weathering.

The results of tensile tests of PP/TH composites as a function of cross-linked tannin content is shown in Figure 3. As can be seen, Young’s modulus increases with increasing TH content. Similar results were described for organic filler-reinforced PP composites [36,37]. It is generally believed that the capability of a composite interface to transfer elastic deformation depends on the interfacial stiffness and static adhesion strength [38]. During a dynamic extrusion process, extensive cross-linking between tannin and hexamine at high temperature formed stiff tannin thermoset particles. These stiff particles contribute to the improvement of Young’s modulus at increasing TH content. Conversely, the crosslinking process appeared to reduce the hydroxyl groups content of the tannin developing a better interaction with hydrophobic PP matrix. Moreover, the rigid TH restricts the chain mobility of the PP matrix yielding a better static adhesion. This phenomenon can be confirmed by the supplementary experimental results (Supplementary Materials Table S1), Young’s moduli of PP combined with uncross-linked tannin (1.41 ± 0.08 GPa) being slightly lower compared with PP alone (1.53 ± 0.08 GPa), while it is 21.5% lower compared to the combination with cross-linked tannin (TH, 1.80 ± 0.05 GPa). Furthermore, the PP/TH composite without MAPP exhibits a lower modulus (Supplementary Materials Table S2, 1.60 ± 0.05 GPa), indicating its positive effect on tensile properties. Without the MAPP compatibilizer, the absence of covalent bonds across the TH-PP matrix interface results in a worse static adhesion strength. 

Figure 3 gives the tensile strengths and elongations of the composites versus TR content, respectively. A decreasing trend can be observed with increasing TH content. Unlike Young’s modulus, tensile strengths measured at larger deformations are much more sensitive to interactions than the modulus, which means it strongly depends on the interfacial adhesion between the filler and the matrix [39]. The tensile strength of composites containing less than 10% TH are slightly lower than PP and quickly decrease in the composite containing more than 15%TH. Similar trends can be found for the elongations results. The TH agglomeration observed under optical microscopy can be an explanation. Conversely, the higher loading of TH would weaken the interfacial adhesion because of the increasing TH-PP matrix interfacial areas. However, a slightly positive effect of MAPP on the tensile strength of a PP/TH composite can be found when comparing it with the PP/TH composite without compatibilizer. The tensile strength and elongation of PP/10TH are respectively 31 ± 1 MPa and 15 ± 2% with MAPP and; 30 ± 1 and 32 ± 10% without MAPP (Supplementary Materials Table S2). This implies that covalent bonds between MAPP and TH may contribute to such an improvement [40,41].

#### 3.1.3. Rheological Behavior

**Figure 4:**
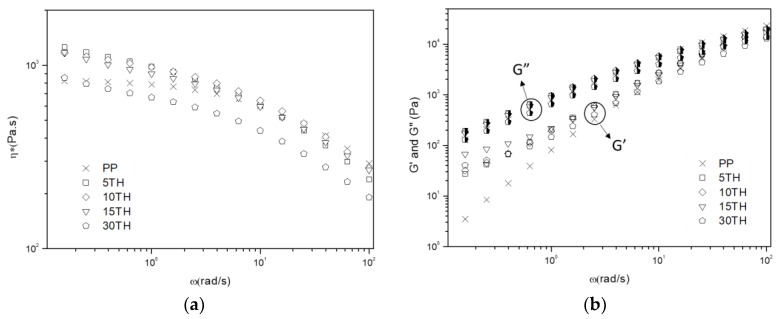
Rheology behavior of PP and PP/TH composites: (**a**) Complex viscosity η*; (**b**) Storage modulus G′ and Loss modulus G″.

The influence of the TH content on the melting rheology behavior of PP was studied by rheometer under the linear viscoelastic region. The complex viscosity (η*), storage modulus (G′) and loss modulus (G″) as a function of frequency (ω) were plotted in Figure 4. In Figure 4a, all curves of η* are observed to decrease with increasing ω, indicating that PP and PP/TH composites exhibit a typical shear-thinning behavior. TH has a positive effect on complex viscosity at low frequency, especially PP containing 5–15% TH. At high ω, η* of PP/TH composites coincide with PP and an increase is observed for PP/TH composites at low η*, replying TH enable a greater resistance to flow. However, 30TH has a lower ω, which suggests that an easier flow tendency caused by weaker interfacial adhesion due to the increase of interfacial areas between TH and PP matrix. A similar trend is also observed in storage modulus (Figure 4b). At low ω, PP/TH composites show a significant increase of G′, which is common in filled polymer system [42,43,44]. This suggests that the presence of cross-linked tannin in the matrix reinforces the internal network structure, enhancing the storage modulus and reduce the deformation degree under a low ω. All studied samples are dominated by the viscous behavior according to loss modulus G″ which is generally higher than the storage modulus G′. However, PP/TH composites present a more solid-like response than PP at low frequency because of the reinforcement of TH in the PP matrix. Unlike tannin-based epoxy resin that behaves like a plasticizer in a PVC matrix [26], cross-linked TH in a PP matrix behaves more like an organic filled PP, for instance, silica reinforced PP [44] and plate-like carbonaceous particles reinforced PP [42].

#### 3.1.4. Crystallinity 

Since PP is a semi-crystalline material, crystallinity has a significant influence on the mechanical properties of PP. In this study, the effect of cross-linked tannin on the crystallinity of PP was characterized by differential scanning calorimetry (DSC). The melting temperature (*T_m_*), melting enthalpy (*H_m_*), and degree of crystallinity (*X_c_*) are presented in Table 1. According to the summarized data, the melting temperature (*T_m_*) of PP was slightly affected by the loading of cross-linked tannin. Like PP-based composites combined with lignin [45], inorganic filler [46], cellulose fiber [6] et al., the crystallinity degree of PP increases with the addition of cross-linked tannin. Thus, it can be concluded that cross-linked tannin can be a nucleating agent for the PP matrix. This nucleation effect might also contribute to the improvement of Young’s modulus.

#### 3.1.5. Thermogravimetric Analyses

**Figure 5:**
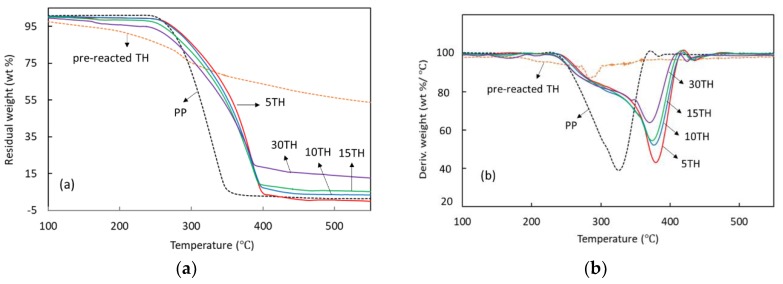
Thermal behaviors of PP and PP/TH composite: (**a**) TGA curves; (**b**) DTG curves.

To better understand the thermal behavior in our environment, the thermal characteristics were determined by Thermogravimetric analyses in an air atmosphere. The weight loss of PP/TH composites as a function of temperature (a) as well as their DTG data (b) are plotted in Figure 5. The weight losses show that degradation of PP and PP/TH composites occurs almost as a one-step process as can be concluded by the presence of only one peak in the DTG curves. A slight weight loss in all the PP/TH composites before the thermal degradation temperature (about 250 °C) is due to either the degradation of small oligomers in TH or the emission of water caused by the crosslinking of the unbound tannin-hexamine component. A general lower degradation speed can be observed in all PP/TH composites, implying a better thermal resistance due to the stabilization of the cross-linked tannin and the restriction of the polymer chains slippage in the presence of cross-linked tannin [47]. It is worth noting that 5TH has the best stabilization performance, followed by 10TH, 15TH and 30TH. This trend might be due to the MAPP compatibilizer, resulting in better compatibility of the 5% cross-linked tannin and PP. Conversely, PP/TH composites present an improvement in char yield when increasing TH content, suggesting a potential flame-retardant property of the cross-linked tannin. Indeed, the char can effectively hamper the oxygen to reach the combustion zone, reducing the combustion rate of polymeric materials.

### 3.2. Anti-UV Performance of Cross-Linked TH

**Figure 6:**
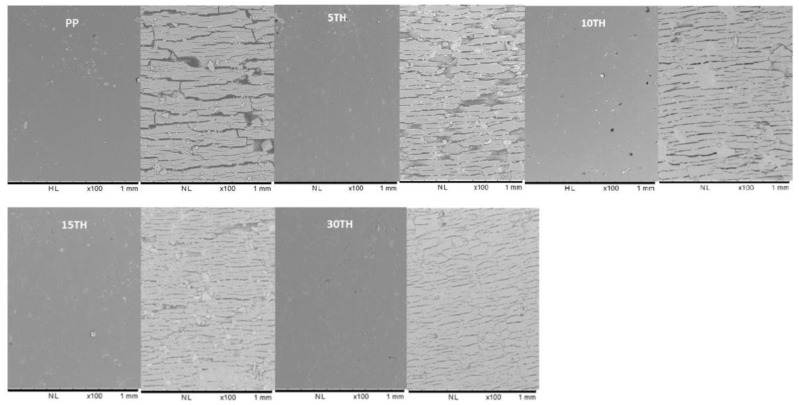
SEM microscopy of PP and PP/TH composites before (left) and after (right) UV weathering.

The degradation of PP caused by sunlight or UV radiation in the air can cause color changes, while also causing surface cracking, and ultimately lower mechanical properties [7]. Figure 6 shows the comparison of microphotographs of unexposed and exposed PP and PP/TH composites characterized by SEM. Both show similarly smooth surfaces without any crack before weathering. However, after one-week UV weathering, surface cracks had appeared in all samples. In the exposed surfaces of PP, large and long cracks mixed with various tiny cracks can be easily observed. Whereas the cracks clearly appeared to be smaller and less severe with an increase of TH content. The cracks were generally found in PP-based composite products in UV exposed surface [47,48], which is caused by the chain scission of the polymer promoted by UV irradiation. The appearance of the cross-linked tannin would indicate that it could prevent photodegradation of the PP matrix.

**Figure 7:**
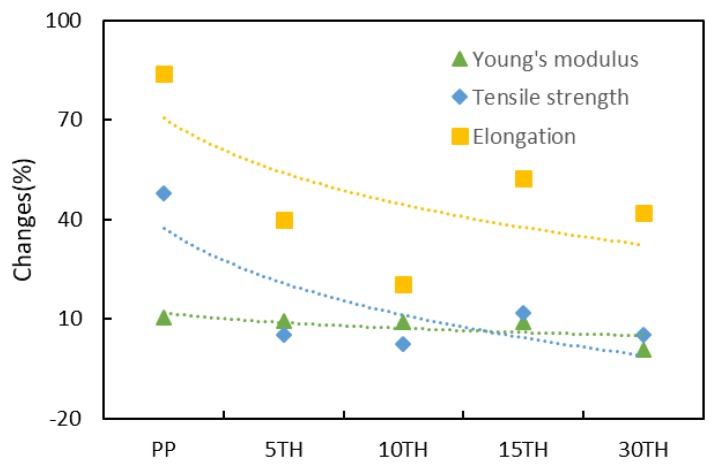
The changes of tensile property on PP and PP/TH composite before and after UV weathering.

The Young’s modulus, tensile strength, elongation of PP and PP/TH composites are presented in Figure 3, and their changes are plotted in Figure 7. According to the report of Badji et al. [47], the frequency and size of cracks have a positive correlation with the mechanical properties. As seen in Figure 3, PP has the worst mechanical performance to resist to large deformations, a significant loss in both tensile strength and elongation being observed after UV weathering. The photodegradation caused by UV irradiation is generally concentrated near the surface. The presence of cracks enhances the permeability to oxygen, resulting in further interior degradation. As a result, PP presents a strong decrease in tensile strength and elongation compared to the sample including cross-linked tannin.

A significant crystallinity decrease of PP after 1-week exposure can be observed, from 34.6% to 25.7% (Table 1) while in presence of TH the crystallinity decrease is clearly lower. The drop of crystallinity is mainly observed for the samples with surface cracks and drop of tensile properties. According to some studies of PP-based composites, an increase in crystallinity can be observed after weathering because the chains scission results in smaller molecules which can undergo recrystallization [9,49]. However, as the chain scission continues, the molecular weight of PP decreases and causes irregular and chemical imperfect recrystallization. Therefore, the less significant changes of crystallinity of PP/TH composites confirm that TH has the ability to hinder chains scission. According to the curing mechanism of tannin-hexamine [17,50,51], the crosslink reaction of TH is mainly dominated by benzylamine bridges between the reactive carbon sites on the flavonoid molecules of tannin and hexamine. As a result radical capacity is promoted due to unsaturated hydroxyl groups. 

**Figure 8:**
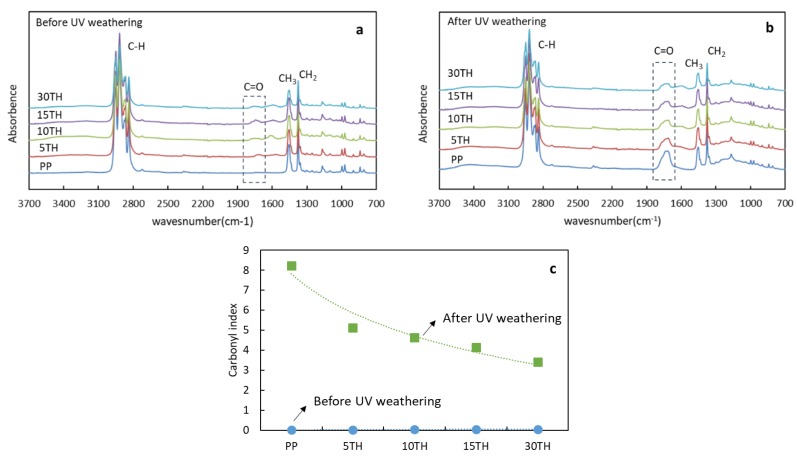
(**a**) FTIR spectra of PP and PP/TH composite before UV weathering (**a**); after UV weathering (**b**) and their carbonyl index (**c**).

The chemical changes of PP and PP/TH composites were characterized by FTIR. Before UV weathering, main chemical bands of PP in the range of 2760–3020 cm^−1^, at 1449 cm^−1^ and 1375 cm^−1^ are visible from Figure 8a. A carbonyl absorption band (1700–1800 cm^−1^) from PP/TH composites attribute to the structure of tannin. In comparison with initial stage, an intensity increase or appearance (PP) of the carbonyl group after UV weathering can be observed in Figure 8b. While a decreasing trend in the intensity of the carbonyl group region with increased addition of cross-linked tannin can be observed, this decrease is confirmed by the changes of carbonyl index from Figure 8c, implying a serious degradation of the PP polymer chains. TH-containing PP instead shows a clear slowdown in degradation due to the formation of hydroperoxides in a lesser extend. Tannins are well known radical scavengers [52] and could be capable to deactivate free radicals by donating a hydrogen atom, preventing the initiation of new radicals in the polymer. In our case, although tannins undergo a dynamic extrusion process of a high temperature in an extruder, its crosslink with hexamine did not consume whole hydroxyl groups. Moreover, the limited residence time in the extruder (approximately 2 min 30 s), compared with more than 5 min for wood bonding [53], and the steric hindrance of tannin might also contribute to an incomplete cross-linked.

**Figure 9:**
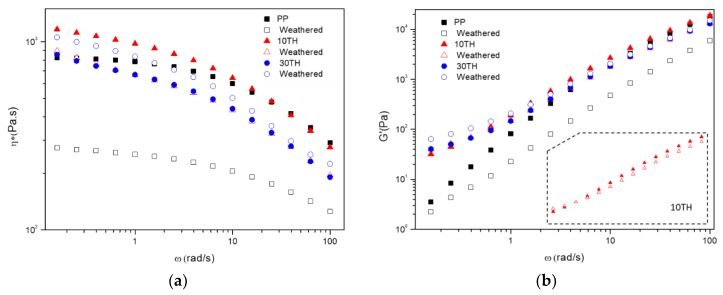
A comparation of (**a**) complex viscosity η* and (**b**) loss modulus G′ of initial and weathered PP, 5TH and 30TH.

In order to better understand how the cross-linked tannin affects PP polymer chains after UV weathering, PP and PP containing 10TH and 30TH were investigated by dynamic rheology, which is a sensitive technique to investigate the structural changes on the weathering degradation process [54]. A comparison of complex viscosity η* and loss modulus G′ between initial and weathered samples is given in Figure 9. The significant drop of η* and G′ for PP suggests a severe chain scission and molecular mass decrease. In agreement with our previous discussion, PP without cross-linked tannin presents severe surface cracks, low crystallinity, and high carbonyl index; therefore, it is much more sensitive to degradation. Whereas, the presence of cross-linked tannin has a lower influence on the melt rheology in both η* and G′, implying a lower chain scission degree. Besides, in those weathered samples, cross-linked tannin might also contribute to the entanglement of degraded PP chain, thus limiting the movement of polymer chains according to G′-ω data. In 10TH and more obviously in 30TH at low frequency, G′ of a weathered sample is slightly higher than the initial one. The more significant increase of η* in 30TH can support this explanation.

## 4. Conclusions

PP-based composite combined with tannin has been successfully processed through dynamic extrusion. In this process, tannin was cross-linked with hexamine in different proportion. The morphology study showed small particles of cross-linked tannin well dispersed in the PP matrix. From Young’s modulus and rheological behaviors of tested samples, cross-linked tannin performed as a stiffness filler and reinforced the PP matrix. In addition, cross-linked tannin had a positive performance towards PP crystallinity and thermal stability. Moreover, when composites were subjected to UV accelerated weathering, PP/TH composites presented a much better performance on photodegradation resistance characterized by fewer surface cracks, lower carbonyl index and less crystallinity decrease, thus slowed down the chain scissions of PP. Furthermore, TH addition to PP can prevent the loss of mechanical properties in physically limiting the mobility of polymer chains. 

## Figures and Tables

**Table 1:** Crystallinity of PP and PP/TH composites before and after UV accelerated weathering.

Sample	Before			After		
	*H_m_*	*X_c_*	*T_m_*	*H_m_*	*X_c_*	*T_m_*
	(J/g)	%	°C	(J/g)	%	°C
PP	−71.6	34.6	165.0	−53.3	25.7	163.3
5TH	−74.9	38.1	166.5	−72.0	36.6	166.8
10TH	−75.6	40.6	164.1	−62.0	33.3	167.6
15TH	−71.3	40.5	164.0	−57.7	32.8	164.7
30TH	−59.1	40.8	164.2	−49.6	34.2	165.1

## Supplementary Materials

The following are available online at http://www.mdpi.com/2073-4360/11/1/102/s1. Figure S1: (a) PP+10% crosslinked tannin; (b) PP+ 30% crosslinked tannin; Table S1: Comparation of tensile property of tannin(T), dynamic crosslinked tannin(TH); Table S2: Comparation of tensile property with or without compatibilizer (MAPP); Figure S2: FTIR spectra of pre-reacted tannin-hexamine (TH); Table S3: Assignment of FT-IR spectra of mimosa tannin and pre-reacted TH.
