# Supplementary material for: Dynamically Cross-Linked Tannin as a Reinforcement of Polypropylene and UV Protection Properties
Source: Polymers (Basel). 2019 Jan 9;11(1):102. doi: 10.3390/polym11010102 (PMC6401781; doi:10.3390/polym11010102)
Supplement: Supplementary file 1 [file polymers-11-00102-s001.pdf]

Dynamically crosslinked tannin as a reinforcement of polypropylene and UV protection properties

Jingjing LIAO [1], Nicolas BROSSE[1]*, Antonio PIZZI[1] and Sandrine HOPPE[2]

Supplementary materials

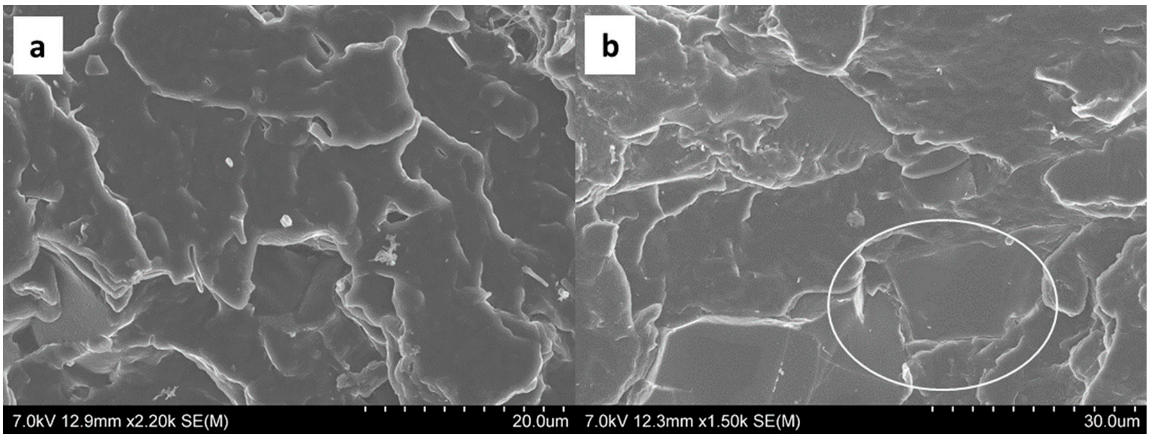

Figure S1 (a)PP+10% crosslinked tannin; (b) PP+ 30% crosslinked tannin

Table S1 Comparation of tensile property of tannin(T), dynamic crosslinked tannin(TH)

Sample	Young's modulus (GPa)	Tensile strength (MPa)	Elongation (%)
PP	1.53±0.08	34±3	52±28
PP+10T+PPMA	1.41±0.08	33±1	21±8
PP+10TH+PPMA	1.80±0.05	31±1	32±10

Table S2 Comparation of tensile property with or without compatibilizer (MAPP)

Sample	Young's modulus (GPa)	Tensile strength (MPa)	Elongation (%)
PP	1.53±0.08	34±3	52±28
PP+10TH	1.60±0.05	30±1	15±2
PP+10TH+MAPP	1.84±0.05	31±1	32±10

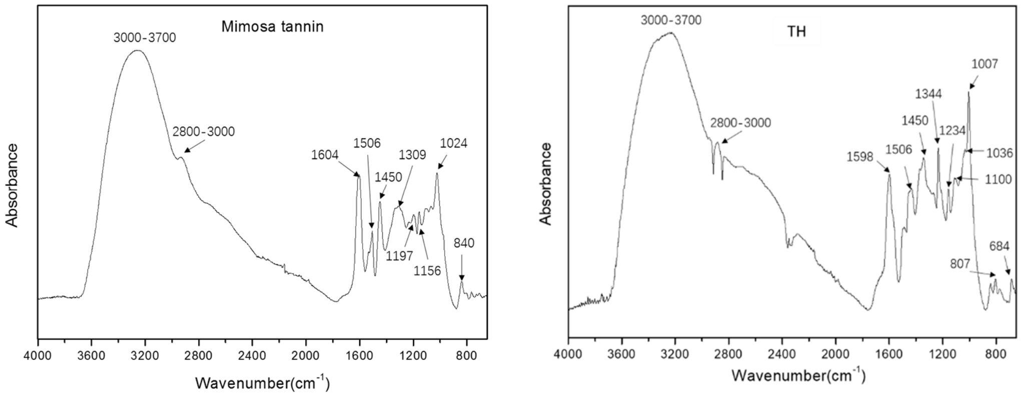

Figure S2 FTIR spectra of pre-reacted tannin-hexamine (TH)

Table S3 Assignment of FT-IR spectra of mimosa tannin and pre-reacted TH

Peak(cm^{-1})	Assignment
3000-3700	O-H stretching of benzene nucleus, methylol groups of tannins
2700-2800	C-H stretching, -CH2- and -CH2OCH2- bridges
1604,1598	C=C aromatic rings stretching
1506	C=C aromatic rings stretching
1450	C=C aromatic rings stretching
1309	C-C frame stretching
1197	C-OH bending
1156	C-O groups of the A-ring resorcinol-like structure
1024	C–H aromatic rings in-plane bending
840	C–H deformation in plane
1344	the C-O stretching of the B-ring of pyrogallic moieties.
1234	antisymmetric deformation of C-O-C in ether groups
1007	C-N groups
807	Deformation vibrations CH bonds
